# Multidetector computed tomography angiography: Application in vertebral artery dissection

**DOI:** 10.4103/0972-2327.78048

**Published:** 2011

**Authors:** Evelyn Teasdale, Peter Zampakis, Celestine Santosh, Saif Razvi

**Affiliations:** Department of Neuroradiology, Institute of Neurological Sciences, Southern General Hospital, 1345 Govan Road, Glasgow G51 4TF, United Kingdom; 1 Department of Neurology, Institute of Neurological Sciences, Southern General Hospital, 1345 Govan Road, Glasgow G51 4TF, United Kingdom

**Keywords:** Multidetector computed tomographic angiography, vertebral artery dissection, stroke, sub-arachnoid hemorrhage

## Abstract

**Background and Purpose:**

Multidetector computed tomography angiography (MDCTA) is a minimally invasive radiological technique providing high-resolution images of the arterial wall and angiographic images of the lumen. We studied the radiological features of vertebral artery dissection (VAD) in a consecutive series of patients investigated for acute stroke and subarachnoid hemorrhage (SAH) in order to confirm and define the diagnostic features of VAD on MDCTA.

**Patients and Methods:**

Review of patients identified prospectively over a 4-year period with VAD assessed by MDCTA was conducted. Radiological features of VAD on MDCTA were reanalyzed utilising previously reported criteria for VAD.

**Results:**

Thirty-five patients (25 males, mean age 49.6 years) with a total of 45 dissected vertebral arteries were reviewed. MDCTA features of VAD included increased wall thickness in 44/45 (97.7%) arteries and increased total vessel diameter in 42/45 arteries (93.3%). All dissected arteries had either lumen stenosis (21/45) or associated segmental occlusion (24/45). An intimal flap was detected in 6/45 (13.3 %) vessels. Twenty-five patients had follow-up imaging, 14/32 vessels returned to normal, 4 showed improvement in stenosis but did not return to normal and 14 demonstrated no change. The majority of non-occluded vessels became normal or displayed improved patency. Only 4/17 occluded arteries demonstrated re-establishment of flow. No adverse effects were recorded.

**Conclusions:**

MDCTA is a safe and reliable technique for the diagnosis of VAD. Increased wall thickness (97.7%) and increased vessel wall diameter (93.3%) were the most frequently observed features.

## Introduction

Vertebral artery dissection (VAD), spontaneous or traumatic, is increasingly recognized as a cause of stroke, especially in younger patients.[1–3] Anticoagulation is often utilized for stenotic/occlusive VAD to prevent secondary thromboembolism.[4] Prompt diagnosis of dissection is important because subsequent stroke may be delayed, and therefore potentially avoided, by early intervention.[5] Stenting over the dissection is an alternative, but as yet, unproven treatment.[6–8]

Digital subtraction catheter angiography (DSA) has been used as the standard technique for VAD diagnosis. However, DSA does not demonstrate vessel wall pathology.[9,10] Diagnosis through DSA, therefore, depends on features found in only 10% of patients (intimal flap, double lumen or dissecting aneurysm) and in most patients is inferred only from indirect features of VAD (irregular stenosis, occlusion or aneurysmal dilatation). Due to DSA’s limitations, non-invasive techniques that visualize the vessel wall, such as Magnetic Resonance Imaging (MRI) with Magnetic Resonance Angiography (MRA) and Multidetector Computed Tomography Angiography (MDCTA), are increasingly utilized.[11–16] In contrast to DSA, MDCTA is widely available and is easily performed after an initial axial CT scan.

Our center has used MDCTA as the first-line radiological tool for arterial assessment in patients presenting with acute stroke and subarachnoid hemorrhage (SAH) since the year 2000. We have applied MDCTA criteria for VAD similar to those initially described by Kurokawa *et al*. and subsequently by Chen *et al*.[11,13]

In this paper, we review patients with VAD studied by MDCTA over a 4-year period, including both spontaneous and traumatic VAD.

## Patients and Methods

Patients in the 4-year study period included the following.

1. Patients with VAD diagnosed on MDCTA and added prospectively to a database for VAD.
2. Patients with VAD diagnosed on review of all radiological requests and review of all scan results of all patients with stroke assessed by MDCTA in the 4-year study period.
3. Patients with suspected but not proven VAD from prior imaging with MR/MRA who had VAD confirmed on MDCTA.
4. Patients with SAH and VAD identified on MDCTA. All such patients had subsequent DSA as a prelude to intravascular treatment or to exclude a missed aneurysmal cause. DSA confirmed MDCTA diagnosis in all these patients.

Our cohort, therefore, included all patients with MDCTA evidence of spontaneous or traumatic VAD in the study period (January 2003–January 2006).

Our radiological observations were reviewed and re-analyzed on the basis of MDCTA criteria for VAD described in two previous series.[13,16]

Clinical features were reviewed retrospectively from case records.

### Multidetector computed tomography angiography technique

Patients were scanned with a quad multidetector helical CT scanner (Philips MX8000, Royal Philips Electronics, the Netherlands). Two MDCTA techniques were employed during the period reviewed. In acute stroke patients who were confused and unable to cooperate, a limited scan from the level of the sixth cervical vertebra to the Circle of Willis was performed. Twenty-one patients were examined in this manner using a 1 mm helix [field of vision (FOV) 180 mm, 0.5 second per rotation, 1.3 mm slice thickness, 0.6 mm table increment (pitch 0.875), 120 kV and 210 mA, 18 second delay from start of injection to start of scan]. A similar protocol was used for those with SAH, but this commenced at the level of the second cervical vertebra. The remaining 14 stroke patients were able to cooperate fully with breathing instructions and so had a complete examination from the aortic arch to the Circle of Willis using a 2.5 mm helix (pitch 0.8, 0.5 second rotation, scan triggered when the contrast density was 150 Hounsfield Units in the ascending aorta). In all patients, 100 ml of non-ionic low-osmolar contrast media [Iobitridol 350 mg L/ml (Xenetix^®^, Laboratoire Guerbet)] was administered via a cubital vein at a rate of 4 ml/second. Patients spent about 20 minutes in the radiology department although the scan acquisition was over in under 30 seconds. Reconstructed images were processed on Philips workstation (MX VIEW). An assessment of the vessels from the axial base data was most relevant with use made of maximum intensity projections (MIP), multiplanar reformations (MPR) and volume rendered three-dimensional (3D) images. These were the same standard post-processing techniques used by the radiologist in the original diagnostic image assessment and were completed within 10–15 minutes.

### Multidetector computed tomography angiography image assessment

**Figure 1:**
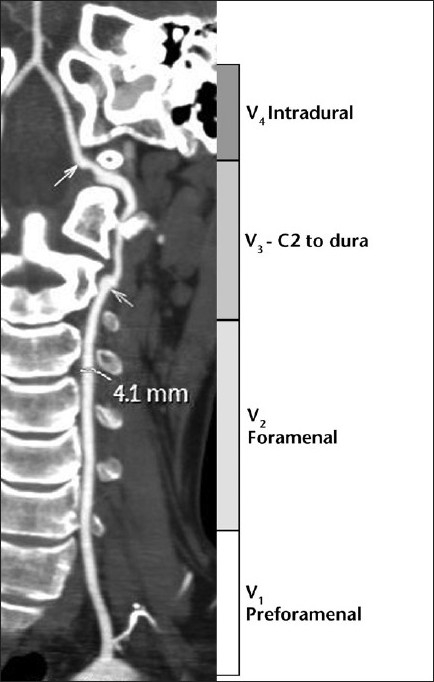
A 6-mm coronal slab maximum intensity projection CT angiogram. The four divisions of the vertebral artery are illustrated. The maximum diameter of the left vertebral artery is measured as 4.1 mm. There is dissection of the V3 segment between the arrows with aneurysm formation at the origin of the dissection and irregular stenosis of the lumen which becomes normal as the artery pierces the dura

All patients had originally been diagnosed as having VAD on the basis of our center’s imaging criteria for VAD on MDCTA. All MDCTA studies were re-assessed by consensus review by two neuroradiologists (CS and ET) experienced in assessing CT angiographic examinations. MDCTA of individual vertebral arteries was analyzed using the standard four anatomical segmentation of the vertebral artery [Figure 1].

**Figure 2:**
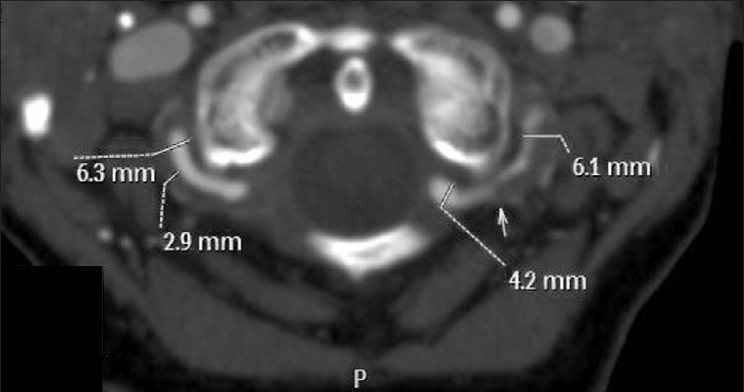
This is an axial 2-mm MIP at the level of C1 of the same patient as in Figure 1. On the left, the total vessel diameter is 6.1 mm which reduces to the normal 4.2 mm diameter just at the dural margin. The shoulder where the dissection ends lies at the level of the arrow. On the right, the total vessel diameter is again increased, the wall of the vessel thickened at 2.9 mm and the vessel occludes at the level of the dura

**Figure 3:**
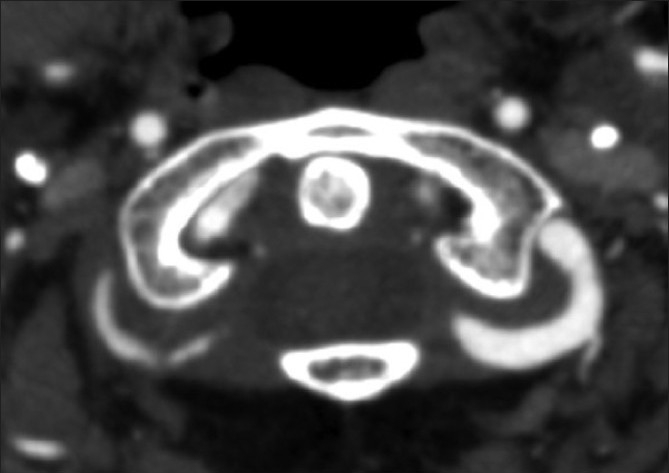
Axial 2 mm MIP at the level of C1.The left vertebral artery is normal. No wall can be identified as is the usual situation. On the right, there is irregular tapered occlusion of the V3 dissected segment. There is also evidence of vessel wall thickening

**Figure 4:**
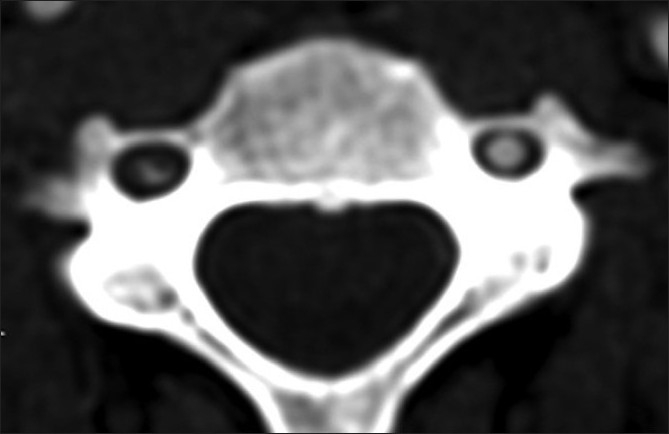
Axial 2 mm MIP at the level of C4. The left vertebral artery is normal. The right shows a lumen compressed on either side by low attenuation filling defect. This is a dissection flap and as the images are scrolled up and down on the workstation, it becomes evident that the dissection flap rotates around the vessel wall

**Figure 5:**
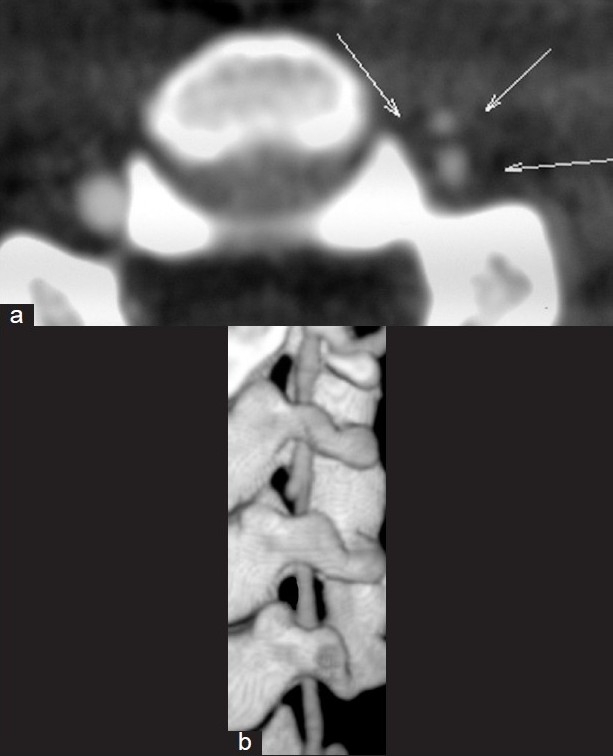
(a) Axial base image. Arrows point to the thickened wall and the pseudoaneurysm lying anterior to the vertebral artery lumen. (b) Volume rendered 3D showing the small pseudoaneurysm at the C3/C4 level

We analyzed the vertebral arteries for the following features:

- Wall thickness and total vessel diameter (comparison with a non-affected segment on the ipsilateral or contralateral artery, taking into account the recognized anatomical differences in size of the vertebral arteries) [Figures 1 and 2]
- Segmental luminal stenosis [Figures 1 and 2]
- Vessel occlusion combined with 1 above (in accordance with the published data and our own observations, vessel occlusion without increased total vessel diameter was diagnosed as embolic occlusion and so excluded from the study group) [Figure 3]
- Intimal flap [Figure 4]
- Double lumen
- Pseudoaneurysm [Figure 5a and b]

The consensus view of CS and ET was recorded.

## Results

**Figure 6:**
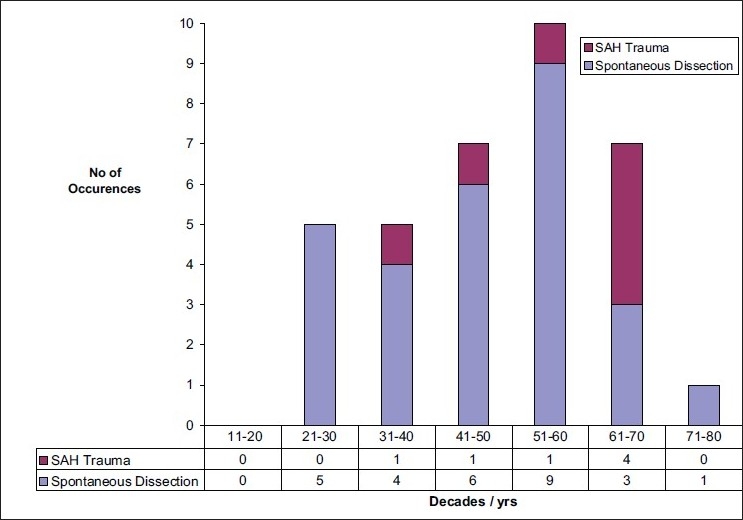
Age distribution in decades: Spontaneous stroke group and total group

Thirty-five patients had VAD confirmed on MDCTA in the 4-year period (45 dissected vertebral arteries, 25 left vertebral artery, average age 49.6 ± 13.7 years, 25 males). Figure 6 shows the age distribution in patients with VAD. 13/28 patients with spontaneous VAD were aged 50 years or above.

31/35 patients were identified from the prospective department database for VAD. 2/35 patients were identified on review of all radiological requests and review of all scan results of stroke patients in the study period. 2/35 patients had initial MRI/MRA, and subsequent MDCTA confirmed VAD in both the patients. In the study period, 651 patients investigated for acute stroke had MDCTA as part of their investigation for stroke. 31/651 patients had VAD (4.76% of stroke). 131/651 patients had posterior circulation stroke. 31/131 (24%) of posterior circulation stroke were therefore associated with VAD. In the remaining patients, 45% had a completely normal MDCTA; 9.7% demonstrated vertebral or basilar atheroma; 8% had vertebral artery occlusion/stenosis without wall thickening as per previous defining criteria for VAD; basilar occlusion/stenosis was found in 6.5%, with posterior cerebral artery occlusion/stenosis in 5.7%, and 1 patient had subclavian steal.

4/35 patients had SAH associated with VAD. DSA confirmed the VAD diagnosis in all four patients. In the study period, 628 patients were investigated for SAH. 4/628 of SAH were therefore associated with VAD.

In total, 6/35 patients had DSA following initial MDCTA (all four with SAH and two others in whom the referring clinician requested DSA). All six DSA studies confirmed VAD.

### Clinical features of vertebral artery dissection

Patient characteristics and clinical features are summarized in Table 1. VAD was a spontaneous event in 32/35 individuals and post-traumatic in three (road traffic accidents). Besides the three with road traffic accidents, two others reported minor trauma prior to presentation. Although other individuals mentioned factors such as odd head positions and exercise, these factors could not be objectively assessed because the distinction between minor trauma and normal daily activities is indistinct. Fifteen patientshad headache, 18 had neck pain, but 8 patients had neither of these preceding admission. Thirteen patients had decreased consciousness, including all four with SAH.

**Table 1:** Clinical features of patients with vertebral artery dissection

Sex	Age (years)	Trauma	Onset	Headache	Neck pain	Conscious level	Horner’s syndrome	Facial paresis and/or sensory loss	Hemi or tetra paresis	Sensory loss in limbs	Dysphagia and/or dysarthria	Comments
M	48	No	Sudden	Yes	Yes	Normal	Yes	No	No	No	No	Associated left internal carotid artery dissection
F	54	No	Sudden	Yes	Yes	Normal	No	Yes	Yes	No	Yes	Right PICA infarction
F	29	No	Sudden	Yes	Yes	Normal	No	No	Yes	Yes	No	Medullary infarction
M	51	No	Sudden	No	No	Normal	No	No	No	Yes	Yes	Left PICA infarction
F	25	No	Sudden	Yes	Yes	Decreased	No	No	No	No	No	Associated internal carotid artery dissection
M	59	No	Sudden	Yes	Yes	Decreased	No	No	No	No	No	Sub-arachnoid hemorrhage
M	45	No	Sudden	Yes	No	Normal	No	No	No	No	No	Right PICA infarction
M	30	No	Gradual	Yes	No	Normal	Yes	Yes	No	Yes	Yes	Bilateral cerebellar infarction
M	52	No	Sudden	Yes	Yes	Normal	No	Yes	No	Yes	Yes	Bilateral cerebellar infarction
F	48	Yes	Sudden	No	No	Decreased	No	No	No	No	Yes	Traffic accident, occipital and left cerebellar infarction
M	36	No	Sudden	Yes	Yes	Normal	Yes	Yes	No	Yes	No	Left PICA infarction
F	29	No	Sudden	No	Yes	Normal	Yes	Yes	No	No	Yes	Right medullary infarction
M	32	No	Gradual	Yes	Yes	Decreased	No	Yes	Yes	Yes	Yes	Locked-in, basilar artery occlusion, pontine infarction
F	62	Yes	Sudden	No	No	Normal	No	No	No	No	Yes	Traffic accident, odontoid peg fracture
M	31	Yes	Sudden	No	No	Decreased	No	No	Yes	Yes	Yes	Cervical cord trauma, bilateral occipital infarction
M	64	No	Gradual	No	No	Normal	No	No	No	No	Yes	Occipital and cerebellar infarction
M	59	No	Gradual	No	Yes	Normal	No	No	Yes	Yes	No	Cervical cord stroke
M	39	No	Gradual	Yes	Yes	Normal	No	Yes	No	No	Yes	Left lateral medullary infarction
M	65	No	Sudden	Yes	Yes	Decreased	No	No	No	No	No	Sub-arachnoid hemorrhage
M	57	No	Sudden	No	No	Decreased	No	Yes	Yes	Yes	Yes	Basilar artery occlusion
F	48	No	Gradual	Yes	No	Normal	No	No	Yes	No	No	Left PICA infarction
M	45	No	Sudden	Yes	No	Decreased	No	Yes	Yes	Yes	No	PICA infarction
M	65	No	Sudden	Yes	No	Decreased	No	No	No	No	No	Sub-arachnoid hemorrhage
M	64	No	Sudden	No	No	Normal	No	Yes	Yes	No	Yes	Top of the basilar artery syndrome
M	50	No	Sudden	No	No	Normal	No	Yes	Yes	No	Yes	Left thalamic and bilateral PICA infarction
M	52	No	Sudden	Yes	No	Normal	No	Yes	Yes	Yes	Yes	Basilar artery occlusion
M	56	No	Gradual	No	No	Decreased	No	No	No	No	No	Right thalamic and right cerebellar infarction with dorsal mid-brain syndrome
M	52	No	Sudden	No	No	Normal	Yes	Yes	Yes	Yes	Yes	Left lateral medullary syndrome
M	73	No	Sudden	No	Yes	Normal	No	No	No	No	No	Scans performed 3 weeks after symptom onset – normal MRI brain
F	30	No	Sudden	Yes	Yes	Normal	No	No	Yes	No	No	Post-partum left vertebral artery dissection
M	33	No	Sudden	Yes	No	Normal	No	No	Yes	Yes	No	Left occipital and thalamic infarction
F	70	No	Sudden	Yes	No	Decreased	No	No	No	No	Yes	Sub-arachnoid hemorrhage
F	70	No	Sudden	Yes	Yes	Decreased	No	No	Yes	Yes	Normal	Left thalamic infarct
M	63	No	Sudden	No	Yes	Normal	Yes	No	No	Yes	Yes	Left occipital infarct, bilateral cerebellar infarcts, giant cell arteritis
M	49	No	Sudden	No	Yes	Decreased	No	Yes	Yes	Yes	Yes	Basilar artery occlusion

### Multidetector computed tomography angiography features of vertebral artery dissection

Quality of acquired MDCTA images was satisfactory in all 35 patients with adequate image contrast. Of the few examinations affected by dental amalgam or swallowing artifact, none rendered the examination nondiagnostic. The results are summarized in Table 2 which includes comparative data from two other studies by Chen *et al*. and Vertinsky *et al*.[13,16]

**Table 2:** Comparison of series of radiological features of vertebral artery dissection on multidetector computed tomography angiography

Radiological feature	Teasdale *et al*. (total 45 arteries) n (%)	Chen *et al*.^1^ (total 19 arteries) n (%)	Vertinsky *et al*.^2^ (total 10 arteries) n (%)
Increase in external diameter	42 (93)	19 (100)	Not reported
Mural thickening	44 (98)	15 (79)	9 (90)
Stenotic dissection	21 (47)	5 (26)	10 (100)
Occlusion + increased external diameter	24 (53)	7 (37)	2 (20)
Aneurysmal dilatation	1 (3)	7 (37)	1 (10)
Intimal flap	6 (17)	0	1 (10)
Double lumen	0	Not reported	Not reported
Bilateral dissection	10 (22)	2 (10)	2 (25)
Associated carotid dissection	2 (4)	0	0
V4	6 (17)	12 (60)	Not reported
V3 + V4	10 (28)	3 (15)	Not reported
V3	7 (20)	3 (15)	Not reported
V3 + V2	8 (22)	Not reported	Not reported
Involving V3	25 (71)	6 (30)	Not reported
V1–V2	10 (28)	1 (5)	Not reported
V1–V4	2 (6)	Not reported	Not reported
Multiple segments	21 (47)	3 (15)	Not reported
CT type	4-slice	4-slice	16-slice
Years of data collection	January 2003–January 2006	July 2000–April 2003	January 2003–March 2007
Number of patients	35	17	8
Mean age (years)	49.6	43	42
Modal decade	6^th^	4^th^	4^th^
Male: Female	25:10	11: 6	3:5

1 Chen *et al*.: Am J Neuroradiology 2004; 25: 769-74

2 Vertinsky *et al*.: Am J Neuroradiology 2008; 29: 1753-60

The MDCTA features of VAD were increased wall thickness in 44/45 arteries and increased total external vessel diameter in 42/45 arteries. All dissected arteries had either multiple lumenal stenosis (21/45) or associated occlusion (24/45). An intimal flap was detected in only 6/45 dissected vessels while the “classic” double lumen sign was not seen in any of our patients and pseudoaneurysm formation was seen in one artery only. Bilateral dissection was detected in 10/35 patients.

The V3 segment was the most commonly affected (24/45) but multiple segments were affected in 21/45. The V1 segment was not included in the 21 patients who had the limited acquisition and only the 3rd and 4th segments were included in those patients with SAH. Two patients had associated dissection of the internal carotid artery; this was unilateral in one individual, but in the other individual all four cervical vessels (both vertebral and both carotid arteries) were dissected.

### Follow-up imaging of arteries

Thirty-two vessels (17 occluded, 15 non-occluded) in 25 patients with ischemic stroke had repeat imaging at an interval, either by MDCTA (17) or MRA (9) (1 patient had both). 14/32 vertebral arteries returned to normal, 4/32 showed improvement in the stenosis and 14/32 did not show a change from the initial imaging.

13/15 of non-occluded vessels became normal or showed improved patency, while only one progressed to occlusion. Only 4/17 occluded arteries showed re-establishment of flow.

## Discussion

This is the largest reported series of patients with VAD assessed by MDCTA.

### Multidetector computed tomography angiography and vertebral artery dissection

Increased total vessel diameter and vessel wall thickening were present in 93.3 and 97.7% of dissected vertebral arteries, respectively. The absence of an increase in total vessel wall diameter in some dissected arteries may be due to subintimal rather than intramural or subadventitial dissection. Stenosis or occlusion of the vessel lumen was therefore present in all dissected vertebral arteries. In contrast, an intimal flap was found in only six dissected vertebral arteries. The combination of an increase in the total diameter of the vessel in association with wall thickening and luminal stenosis or occlusion is therefore highly suggestive of VAD.

The differential diagnosis of such vessel appearances includes atheroma, but this usually affects multiple vessels, the origins and V2 segments of the vertebral artery, but it is an intimal disease and so it does not increase the total vessel diameter. Vessel wall thickening may also be found in Takayasu arteritis, Behcet disease and giant cell arteritis. These disorders are rare, more frequently affect the carotid arteries and must be considered in appropriate clinical circumstances.[17]

Although our results appear to concur with the observations made by Chen *et al*., only one patient in our series had aneurysmal dissection as compared to their study which may reflect differences in the populations studied. Bilateral VAD was detected in 25% of our patients as compared to 12–60% reported in the prior series.[10,13,18] Multiple vessel involvement was not limited to those with trauma and is recognized occurrence thought to relate to an underlying connective tissue problem.

In our series, MDCTA did not include the arch of the aorta in 21 patients. It was therefore not possible to confirm or exclude dissection involving or limited to the V1 segment, potentially reducing detection of multiple-level VAD. In addition, there may have been potential failure to identify patients with isolated V1 dissection in a proportion of patients in the study period.

Modern CT allows assessment of the entire VA and the combination of base image, MPR and MIP analysis gives access to the complete VA unaffected by any theoretical artifact from its small size or bony confines. VAD could be underestimated by MDCTA where there is occlusion without evidence of an increased overall vessel diameter such as may occur with purely subintimal dissection. Overestimation is unlikely if the defining characteristics as described earlier are applied.

### Multidetector computed tomography angiography in comparison to digital subtraction catheter angiography in vertebral artery dissection

DSA when performed in our patients (six patients) confirmed the MDCTA diagnosis in all. In Chen *et al*.’s previous comparison between DSA and MDCTA in patients with stroke, all 17 patients diagnosed with VAD on DSA had vessel wall thickening. Their reported sensitivity of MDCTA in detecting VAD was 100% with a specificity of 98%, with positive and negative predictive values of 95 and 100%, respectively. We did not undertake a systematic direct comparison of MDCTA with other radiological modalities because MDCTA became the established initial angiographic method in the year 2000 and it would have been unethical to carry out DSA purely for the purposes of comparison. We believe that DSA no longer has any additional role in the diagnosis of VAD and indeed may even be contraindicated because of the possibility of further vessel damage. However, DSA is essential if SAH is the presenting feature or if there is distal basilar occlusion when vascular interventional treatment may be necessary.

### Multidetector computed tomography angiography in comparison to magnetic resonance imaging / magnetic resonance angiography in vertebral artery dissection

A recent study has suggested that MDCTA is more accurate in the diagnosis of VAD than MRI/MRA due to the visualization of more diagnostic features.[16]

MDCTA has other practical advantages such as rapid acquisition of information in obtunded or un-cooperative patients, and patients in whom MRI is contraindicated or impossible because of involuntary movement. All standard resuscitative measures can safely be applied to patients in CT unlike the additional restrictions with MRI.

MRI can be useful in confirming hemorrhage in the wall of a dissected vessel. However, disadvantages include its limited spatial resolution, relatively limited area coverage, and the risk of false positive results due to flow related high signal in the venous plexus surrounding the vertebral artery or slow flow within an artery.[14,19,20] In contrast, the high spatial resolution and intrinsic tissue attenuation detected by MDCTA enables the differentiation of intramural clot from the contrast-enhanced residual lumen of a vessel, and the detection of calcific atherosclerotic and vaso-occlusive disease of cervical arteries.[13]

We currently use a 64-slice CT and its improved temporal resolution has allowed us to scan all patients, irrespective of their ability to cooperate, from the arch to above the Circle of Willis. The improved spatial resolution has less effect as the diagnosis of VAD is based on the acquired axial slice. However, MIP and other vascular reconstructions are superior and have made the diagnosis of VAD at least subjectively easier for the radiologist.

The ability of MDCTA to simultaneously demonstrate and evaluate pathology throughout the length of all the cervical arteries in one 16-second (4 slice CT) to one 4-second (64 slice CT) examination is an additional advantage over MR, especially so when dissection may be clinically silent and not suspected.

### Multidetector computed tomography angiography and subarachnoid hemorrhage

It has been suggested that MDCTA can replace DSA in the initial assessment of patients with SAH.[21] Although non-aneurysmal VAD is a rare cause of SAH (0.63% in this study, and also, 0.63% in a previous report using DSA), accurate diagnosis is important because early re-bleeding is common, especially in those dissections associated with a pseudo aneurysm (31%) with a high mortality if VAD is left untreated.[8] Ramgren *et al*. reported 5 deaths in 14 patients treated conservatively from a total of 9 deaths in the 29 patients of their series.[8]

In our center, MDCTA is routinely performed as initial assessment of patients suspected of having SAH. If DSA was the initial tool at our center, dissection could potentially be misinterpreted as vasospasm or iatrogenic dissection, potentially delaying correct diagnosis and management.

### Vessel recanalization

Previous follow-up studies of VAD indicate that 63–88% of narrowed arteries demonstrate improvement in patency.[10,22,23] In our series, 86.6% of stenosed vessels showed normal or improved patency but only 23.5% of occluded VAD recanalized. These observations suggest the potential application of MDCTA in predicting vessel recanalization.

### Vertebral artery dissection and stroke

In our series, 4.7% of patients with stroke had VAD as cause of their stroke. This is much higher than that reported in the literature. This may suggest that VAD was more frequent in our study population or that the use of MDCTA in patients with stroke enables improved detection of VAD. Our results also show that stroke due to VAD is often found in the elderly. Twelve of 35 individuals had evidence of secondary embolic occlusion of distal vessels [Table 1].

## Conclusions

In conclusion, our series confirms the MDCTA features of VAD and demonstrates that MDCTA can reliably and safely demonstrate the direct and indirect features of VAD. The routine use of MDCTA in stroke increases detection of VAD. MDCTA, therefore, promotes early diagnosis of VAD, facilitating prompt initiation of appropriate management.
